# Body image in breast cancer survivors: Age-moderated effects of treatment-induced menopause and fertility concerns

**DOI:** 10.1016/j.breast.2025.104512

**Published:** 2025-06-05

**Authors:** Oshri Saar Sheffi, Shani Paluch–Shimon, Gil Goldzwieg, Shiran Klein Rotchild, Michal Braun

**Affiliations:** a Breast Oncology Unit, Hadassah University Hospital, Jerusalem, Israel; b School of Behavioral Sciences, Tel-Aviv-Yaffo Academic College, Tel-Aviv, Israel; c Faculty of Medicine, Hebrew University, Jerusalem, Israel

**Keywords:** Breast cancer, Body image, Endocrine therapy, Menopause, Fertility, Young survivors

## Abstract

**Purpose:**

Hormone receptor-positive breast cancer survivors undergoing endocrine therapy face significant body image challenges. This study aimed to examine differences in body image between hormone receptor-positive breast cancer survivors receiving endocrine therapy and unaffected, healthy women. Specifically, we investigated whether treatment-induced menopausal symptoms and fertility concerns mediate the relationship between breast cancer survivorship and body image. Additionally, we explored whether age moderates these mediation effects, assessing whether the strength of these indirect relationships varies across different age groups.

**Methods:**

121 hormone receptor-positive breast cancer survivors and 114 healthy women completed a sociodemographic questionnaire, the Body Image Scale (BIS), the Menopausal Rating Scale (MRS), and the Reproductive Concerns Scale (RCS). Breast cancer survivors also completed a medical data questionnaire.

**Results:**

Hormone receptor-positive breast cancer survivors reported significantly higher levels of negative body image, menopausal symptoms, and fertility concerns compared to healthy women. Moderated mediation analyses revealed that both menopausal symptoms and fertility concerns mediated the relationship between breast cancer and body image, with age moderating these relationships. The indirect effects were stronger among younger women and diminished with age. The mediating effect through menopausal symptoms was particularly pronounced for somatic and urogenital symptoms.

**Conclusions:**

These results highlight the significant role of treatment-induced menopausal symptoms and fertility concerns in shaping body image among young hormone receptor-positive breast cancer survivors. These young women should be provided with targeted information and interventions that will help them cope with treatment-related side effects and maintain positive body image as part of the recovery process.

## Highlights

• Young BC survivors report significantly worse body image than healthy women.
• Menopausal symptoms mediate the relationship between BC survivors and body image.
• Fertility concerns mediate the relationship between BC survivors and body image.
• Age moderates these effects, with stronger impacts observed in younger survivors.

## 1 Introduction

Breast cancer (BC) is the most prevalent malignant tumors affecting women globally [1]. Up to 25 % of new diagnoses are in women under 50 and 6–7 % in women under the age of 40 [2]. Among women aged 0–39, BC is the leading cause of cancer-related deaths, with approximately 44,840 deaths globally in 2018 [3]. A substantial proportion of these young patients are diagnosed with hormone receptor-positive (HR+) tumors, requiring 5–10 years of endocrine therapy [4]. While advances in targeted therapies have improved survival rates - from 84 % to 90 % in 5-year survival between 1996-2000 and 2011–2015 in Israel [5], research indicates that BC survivors, defined as women who have completed their primary treatments - surgery, radiation therapy and chemotherapy [6] continue to face long-term psychological challenges related to treatment-induced bodily changes, premature menopause and fertility concern [4,7,8].

Body image is a multidimensional concept referring to the mental picture individuals form of their own body, encompassing perceptions, thoughts and feelings about their physical appearance [9]. It is a crucial aspect of an individual's self-concept and psychological well-being, influencing self-esteem, social interactions, and overall quality of life [10]. Research consistently demonstrates that women treated for BC experience greater body image disturbances compared to healthy women [11]. While numerous studies have explored the impact of different surgical approaches on body image, yielding mixed results [12,13], less attention has been given to the potential long-term effects of endocrine therapies on body image. Endocrine therapies, which are recommended for several years post-surgery, may induce long-term physical changes including weight gain and menopausal-like symptoms, potentially having a significant impact on body image [14]. Age appears to play a significant role, with younger women being particularly vulnerable to this body image disturbance, possibly due to the unexpected nature of these bodily changes and their implications for fertility and feminine identity [11,14,15]. These age-related differences, emerging during a life phase focused on career development, family responsibilities, and childcare, suggest that age influences the strength of relationship between treatment effects and body image, with effects being more pronounced in younger women compared to those in later life stages [16]. Within these treatment-related effects, menopausal symptoms present a unique challenge for young BC survivors.

Treatment-induced menopause in BC survivors differs significantly from natural menopause, which typically occurs between ages 45–56 [17]. While natural menopause involves gradual hormonal changes, endocrine therapy in HR + BC causes an abrupt onset of menopausal symptoms which may include hot flashes, night sweats, vaginal dryness, decrease in libido, dyspareunia, sleep disturbances, and changes in sexual function through medications like Tamoxifen, aromatase inhibitors and GnRH analogues which block estrogen effects or decrease its production [18]. This sudden transition can be particularly challenging for young survivors, who experience symptoms without the psychological preparation that accompanies natural aging. Research indicates that young survivors report significant physical and psychological effects, with studies documenting high rates of body image concerns (30–67 %) alongside other symptoms like decreased libido (23–64 %) and dyspareunia (35–38 %) [19]. For younger women, this premature treatment-induced menopause affects both their body image and reproductive function, creating a complex challenge as they grapple with these unexpected physical and reproductive changes [20].

The impact of compromised reproductive potential emerges as another significant challenge for young BC survivors, affecting both treatment decisions and psychological well-being [21]. These fertility concerns extend beyond reproductive potential, affecting survivors' sense of self and femininity, with many reporting a sense of disconnection from their bodies, perceiving them as fundamentally altered or damaged [22]. While recent research, particularly the POSITIVE study, has demonstrated that temporary interruption of endocrine therapy for pregnancy does not increase short-term recurrence risk [23], the psychological impact of potential infertility continues to affect young survivors' physical self-perceptions. This impact is particularly evident among women who desire future pregnancies but remain uncertain about their fertility status, who report not only higher levels of anxiety and depression, but also more negative body image perceptions compared to those with clarity about their reproductive potential [20].

While previous research has established that BC and its treatments can negatively impact body image, this study extends prior work by identifying specific psychological and physiological pathways that contributes to the effect of BC on body image, particularly in young survivors. By integrating both mediation and moderation, we aim to clarify how menopausal symptoms and fertility concerns interact with age to shape body image perceptions. Treatment induced menopausal symptoms and fertility concerns are hypothesized to mediate this relationship, and age is explored as a moderating factor influencing the strength of these indirect effects. We hypothesized that BC survivors would report a more negative body image than healthy women, with menopausal symptoms and fertility concerns mediating this relationship. We further hypothesized that age would moderate these relationships, with younger survivors showing stronger associations between these factors.

## 2 Method

### 2.1 Participants

A total of 235 women (by self-identification) participated in this study: 121 HR + BC survivors treated with endocrine therapy for at least six months, and 114 healthy women. Inclusion criteria were Hebrew speakers above the age of 18. Exclusion criteria included: non-hormonal BC diagnoses, other types of cancer, chronic diseases, and incomplete responses (more than 25 % missing data).

BC survivors were recruited through social media networks and the oncology department at Shaarei Zedek Medical Center, Israel. Healthy women were recruited through social media networks using snowball sampling. Data collection occurred between 2020 and 2024.

The study received approval from the Ethics Committee of Tel Aviv Jaffa College (approval no. 2020050) and the Helsinki Committee of Shaarei Zedek Hospital (approval no. szmc-0302-20).

### 2.2 Measurements

#### 2.2.1 Body image

The Body Image Scale questionnaire (BIS) [24] was used to assess body image. This 10-item questionnaire explores emotional (feelings of femininity and attractiveness), behavioral (discomfort when viewing oneself), and cognitive (satisfaction with appearance) aspects of body image. Participants rated their experiences over the past week on a 4-point scale from 0 (not at all) to 3 (very much). Higher total scores indicate greater body image disturbance. The scale demonstrated high reliability (Cronbach's α = .94).

#### 2.2.2 Menopausal symptoms

The Menopausal Rating Scale (MRS) questionnaire [25] is an 11-item scale assessing menopausal symptoms across three dimensions: psychological (e.g., depressive mood, irritability), somatic-vegetative (e.g., hot flashes, sleep problems), and urogenital (e.g., sexual problems, bladder problems). Higher scores indicate greater symptom severity. The scale was translated to Hebrew by a team of bilingual psychologists using a standard forward-backward procedure. The subscales showed good reliability (psychological: α = .86; somatic: α = .79; urogenital: α = .65), with high overall reliability (α = .89).

#### 2.2.3 Fertility concerns

The Fertility Concerns Scale (RCS) questionnaire [26] was specifically developed to assess fertility concerns among cancer patients. This 14-item scale evaluates feelings and concerns about pregnancy, fertility, and reproduction during the previous month. Higher scores indicate greater fertility concerns. The scale demonstrated good reliability (α = .82).

#### 2.2.4 Socio-demographic questionnaire and medical information

Participants completed a questionnaire collecting sociodemographic information including age, religious affiliation, education level, marital status, and employment status. BC survivors additionally provided medical information including age at diagnosis, cancer stage, and details about treatments received (type of surgery, chemotherapy, radiation therapy, biological therapy (e.g., Herceptin) and fertility preservation procedures).

#### 2.2.5 Statistical analysis

Data analysis was conducted using SPSS Version 25 (SPSS, Chicago, IL), supplemented with PROCESS Macro [27]. Descriptive statistics were used to characterize and compare between BC survivors and healthy women. To test our hypotheses, we employed moderated mediation analyses (Model 7, Hayes), examining the indirect effect of group status on body image through mediators (menopausal symptoms and fertility concerns), with age as a moderator. All analyses were conducted at a 95 % significance level.

## 3 Results

### 3.1 Sociodemographic variables

Descriptive statistics for sociodemographic and medical variables are presented in Table 1, Table 2. The participants (mean age = 47.2 ± 12.52, range: healthy women 24–74 years, BC survivors 25–78 years) were predominantly married/in a relationship, secular, well-educated, and employed. Significant group differences were found in age, number of children, and religiosity, with BC survivors being older, having more children, and being more religious. However, age showed no significant correlation with body image, while both religiousness (r = .176, p < .05) and number of children (r = .138, p < .05) showed weak but significant correlations. These differences align with Israeli demographic trends linking religiosity with larger family size. Among BC survivors, most had stage II cancer at diagnosis, underwent lumpectomy, and received radiation therapy. The mean time since diagnosis was 5.45 years (SD = 6.56), with the majority (59.2 %) diagnosed within the past 4 years. Notably, the majority did not report fertility problems and had not undergone fertility preservation procedures.

**Table 1:** Sociodemographic variables.

Sociodemographic characteristics	Breast cancer survivors	Healthy women	Comparison			
	Mean (SD)/N(%)	Mean (SD)/N(%)	x2(3)	p	t(233)	p
Age	49.67 (10.55)	44.77(14.93)			2.99	.003
Number of children	2.99 (1.34)	1.43 (1.48)			2.54	.001>
Years of education	15.73 (3.17)	15.92 (2.31)			−530.	.597
Marital status			3.13	.208		
Single	12 (9.9)	18 (15.78)				
In a relationship/married	89 (73.55)	84 (73.68)				
Separate/divorced	20 (16.52)	12 (10.52)				
Religiousness			26.60	001.>		
Secular	74 (61.15)	102 (89.47)				
Religious	37 (30.57)	10 (8.77)				
Ultraorthodox	8 (6.61)	2 (1.75)				
Missing responses	2 (1.67)					
Occupational status			5.52	.137		
Full-time job	52 (42.97)	65 (57.01)				
Part-time job	35 (28.92)	28 (24.56)				
Unemployed	23 (19)	16 (14.03)				
Other	11 (9.09)	5 (4.38)

**Table 2:** Medical Characteristics—breast cancer group.

Medical Date		N(%)
Cancer stage at diagnosis	I	39(32.23)
	II	54 (44.62)
	III	28 (23.14)
Surgery type	Lumpectomy	70 (57.85)
	Mastectomy	42 (34.71)
	Bilateral Mastectomy	9 (7.43)
Treatment type	Neoadjuvant chemotherapy	44 (36.36)
	Adjuvant chemotherapy	43 (35.53)
	Radiation	91 (75.20)
	Biological therapy	37 (30.57)
BRCA gene carrier		15 (12.39)
Self-reported fertility difficulties		13 (10.74)
Fertility conservation for women under 42 years of age at diagnosis		20 (22.2)

### 3.2 Group differences in main study variables

Independent t-tests revealed significant differences between groups across all main study variables. Given that BC survivors were significantly older (M = 49.67, SD = 10.55) than healthy women (M = 44.77, SD = 14.93), t(206.51) = −2.964, p = .003, analyses of covariance (ANCOVA) were conducted with age as a covariate. Results revealed that BC survivors had significantly higher levels of negative body image (M = 26.92, SD = 8.33) compared to healthy women (M = 13.24, SD = 3.99), controlling for age, F(1,231) = 231.87, p < .001, partial η^2^ = .501. Similarly, BC survivors showed significantly higher levels of menopausal symptoms (M = 29.43, SD = 8.28) compared to healthy women (M = 17.56, SD = 5.45), controlling for age, F(1,231) = 159.03, p < .001, partial η^2^ = .408. BC survivors also reported significantly higher levels of fertility concerns (M = 28.52, SD = 11.07) compared to healthy women (M = 22.33, SD = 4.63), controlling for age, F(1,231) = 26.53, p < .001, partial η^2^ = .103.

### 3.3 Interrelationships between the research variables

Analysis of correlations revealed distinct patterns of associations between the study variables for each group (see Table 3). Among BC survivors, body image showed significant positive correlations with both menopausal symptoms (r = .429, p < .01) and fertility concerns (r = .342, p < .01). All menopausal symptom subscales were significantly associated with body image: psychological (r = .502, p < .01), urogenital (r = .219, p < .01), and somatic-vegetative (r = .331, p < .01). In contrast, among healthy women, only somatic-vegetative symptoms correlated with body image (r = .339, p < .01), while psychological (r = .082, p > .05) and urogenital symptoms (r = .100, p > .05) showed no significant associations. No significant correlation was found between fertility concerns and body image in healthy women (r = −.147, p > .05). Additionally, no significant association was found between time since diagnosis and body image among BC survivors (r = .044, p > .05).

**Table 3:** Correlations between the main variables, by group: Breast cancer survivors (in bold) vs. healthy women (not bold).

	MRS	RCS	BIS	MRS-P	MRS-O	MRS-S
Menopausal symptoms (MRS)		**.401**^b^ .031	**.429**^b^ .264^b^	**.870**^b^ .828^b^	**.794**^b^ .700^b^	**.839**^b^ .672^b^
Fertility concerns (RCS)	.**401**^b^ .031		**.342**^b^ −.147	**.419**^b^ .403^b^	**.313**^b^ .011	**.261**^b^ −.050
Body image (BIS)	**.429**^b^ .264^b^	**.342**^b^ −.147		**.502**^b^ .082	**.2119**^a^ .100	**.331**^b^ .339^b^
Psychological symptoms of menopause (MRS-P)	**.870**^b^ .828^b^	**.419**^b^ .403^b^	**.502**^b^ .082		**.555**^b^ .435^b^	**.575**^b^ .254^a^
Urogenital symptoms of menopause (MRS-O)	**.794**^b^ .700^b^	**.313**^b^ .011	**.219**^a^ .100	**.555**^b^ .435^b^		**.514**^b^ **.**272**∗∗**
Somatic-vegetative symptoms of menopause (MRS-S)	.839^b^ .672^b^	.261^b^ −.050	.331^b^ .339^b^	.575^b^ .254^a^	.514^b^ .272^b^	

Bold, breast cancer survivors; not bold, healthy women.

a Correlation is significant at the .05 level (2-tailed).

b Correlation is significant at the .01 level (2-tailed).

### 3.4 Moderated mediation analysis

#### 3.4.1 Menopausal symptom model

**Fig. 1:**
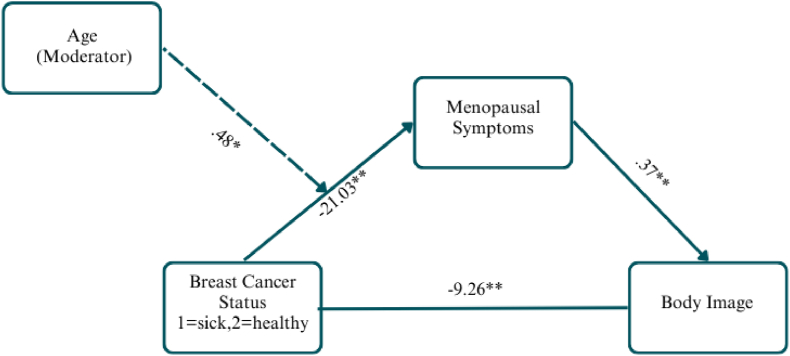
MRS model Unstandardized regression coefficients shown. Menopausal symptoms mediate the relationship between breast cancer status and body image. Age moderates the indirect effect, with stronger effects in younger women.

The overall moderated mediation model examining the effect of group status (BC survivors vs. healthy women) on body image through menopausal symptoms, with age as a moderator, was significant (F(2,219) = 155.97, R^2^ = 58.75 %, p < .01) with a significant direct effect between group status and body image (b = −9.26, SE = 1.09, t = −8.54, p < .01). The index of moderated mediation was significant (index = .1922, BootSE = .0787, 95 % CI: .0379, .3465), indicating that age moderated the indirect effect. This indirect effect decreased with age. (see Fig. 1).

Further analysis of specific symptom subscales revealed significant moderated mediation for both urogenital symptoms (index = .0415, BootSE = .0271, 95 % CI: .0014, .1030) and somatic symptoms (index = .0556, BootSE = .0349, 95 % CI: .0010, .1362), with stronger effects in younger women. However, psychological symptoms showed no significant age moderation (index = .0182, BootSE = .0368, 95 % CI: .0506, .0928).

### 3.5 Fertility concerns model

**Fig. 2:**
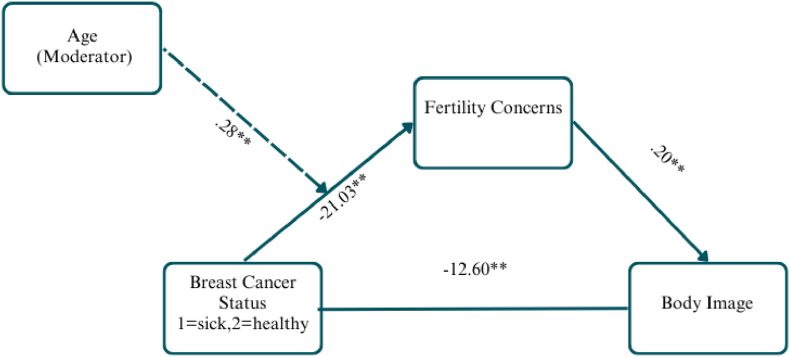
RCS model Unstandardized regression coefficients shown. Fertility concerns mediate the relationship between breast cancer status and body image. Age moderates the indirect effect, with stronger effects in younger women.

The fertility concerns model was significant (F(2,218) = 142.46, R^2^ = 56.65 %, p < .01), with a significant direct effect (b = −12.60, SE = .90, t = −13.97, p < .01). Age significantly moderated the relationship between group status and fertility concerns (b = .2846, SE = .0901, t = 3.1581, p < .01), with stronger indirect effects in younger women. (see Fig. 2).

## 4 Discussion

The objective of this study was to explore differences in body image between young HR + BC survivors and healthy women, while examining the mediating roles of treatment-induced menopausal symptoms and fertility concerns. Our findings revealed that BC survivors reported significantly more negative body image compared to healthy women. Moreover, menopausal symptoms and fertility concerns mediated the relationship between BC survivorship and body image, with age moderating these relationships. The indirect effects were stronger among younger women and diminished with age, highlighting the unique vulnerability of young survivors to body image disturbances. Importantly, body image disturbances persisted regardless of time since diagnosis, suggesting these challenges remain stable throughout the post-treatment phase.

Our findings regarding elevated body image concerns among BC survivors align with previous research [11,28]. However, this study extends previous findings by identifying specific mechanisms through which BC treatment affects body image in young survivors. The mediating role of menopausal symptoms proved particularly significant, with both urogenital and somatic symptoms showing stronger effects in younger women. This finding suggests that the abrupt onset of menopausal symptoms due to hormonal treatment creates a more profound impact when experienced prematurely, potentially due to the psychological unpreparedness for bodily changes at a young age, the discrepancy it creates between survivors' experiences and those of their peers, and the sudden shift in self-identity during young adulthood.

Similarly, fertility concerns emerged as a significant mediator between BC survivorship and body image disturbances, with stronger effects among younger women. This finding highlights how reproductive concerns extend beyond the physical ability to bear children aspects to influence body perception and feminine identity. For this reason, even those who have completed their families prior to diagnosis may experience significant fertility-related distress, as these concerns represent deeper symbolic aspects of loss and feminine identity rather than merely functional reproductive needs [22]. Young survivors face unique challenges as they confront the disruption of their reproductive timeline and life plans, particularly during a period when their peers are actively engaged in family planning [29].

Beyond these specific mechanisms, our findings point to a broader issue in BC care and recovery. Our study results reveal a notable disparity between the medical classification of young HR + BC survivors and their subjective experiences. Although medically classified as 'recovered', these individuals continue to grapple with substantial physical and psychological challenges, many of which are specifically related to their ongoing endocrine therapy [7,16]. This gap is particularly pronounced in younger women, who may struggle to reconcile their status as 'survivors' with the ongoing impact of treatment on their body image and fertility [30,31]. These findings suggest the need for a more refined conceptualization of 'survivorship' that recognizes the long-term, multifaceted nature of BC recovery, particularly for young women whose treatment-related challenges intersect with developmental life stages and expectations.

In addition to these findings, our study revealed a notably low rate of fertility preservation among young BC survivors, with only 20 participants (22.2 % of those under 42 years at diagnosis) reporting having undergone fertility preservation procedures even though over 70 % received (neo)-adjuvant chemotherapy. This finding is particularly concerning when considering the ESO-ESMO fifth international consensus guidelines for BC in young women (BCY5), which strongly recommend discussing fertility issues with all young BC patients before any fertility-impairing treatment [32]. The gap between these guidelines and clinical practice is further highlighted by research showing that only approximately 50 % of female cancer survivors receive reproductive health counseling [33]. This discrepancy between recommendations and implementation raises significant concerns about fertility preservation protocols in clinical practice, especially given that fertility counseling has been associated with better quality of life outcomes and reduced regret among survivors [34]. This gap is particularly noteworthy in the Israeli cultural context, where fertility and family planning hold significant societal importance, with one of the highest fertility rates among developed nations, even among secular families.

While the study offers significant insights, several limitations should be acknowledged. First, the study did not include a control group of BC survivors who had not been treated with endocrine therapy, limiting our ability to isolate the specific effects of hormonal treatment on body image. Future research comparing different treatment protocols could help isolate these specific effects. Second, the cross-sectional nature of the study precludes causal inferences and the detection of changes over time. Longitudinal studies are needed to examine the long-term impact of BC treatment on body image, menopausal symptoms, and fertility concerns. Finally, the use of convenience sampling through social media and snowball methods potentially limits generalizability and may have resulted in more homogeneous samples.

Our findings demonstrate that treatment-induced menopausal symptoms and fertility concerns significantly impact young BC survivors' body image, advancing both clinical understanding and theoretical frameworks. By identifying specific mediating mechanisms, this study moves beyond documenting body image disturbance to explaining its underlying processes. Similarly, while previous research identified age as a risk factor, our results explain this relationship by demonstrating how younger women's premature exposure to menopausal symptoms and disrupted fertility plans amplifies body image concerns. The differential impacts of psychological, somatic, and urogenital symptoms on body image suggest that menopausal symptoms should be viewed as distinct components rather than a single unified experience, each requiring targeted intervention approaches. Furthermore, our findings challenge the assumption that body image concerns naturally diminish post-treatment, indicating persistent effects throughout hormonal therapy. These theoretical insights inform clinical interventions, indicating the need for pre-treatment fertility counseling, age-specific support groups, and long-term psychological support tailored to specific symptom profiles. Healthcare providers should integrate these targeted approaches both at diagnosis and throughout the extended survivorship period, particularly focusing on younger women who show heightened vulnerability to these combined challenges.

## 5 Conclusions

This study demonstrates the significant role of treatment-induced menopausal symptoms and fertility concerns in shaping body image among young HR + BC survivors. Age emerged as a significant moderator of these relationships, with stronger mediating effects observed among younger survivors. These findings underscore the importance of implementing targeted, age-specific interventions addressing menopausal symptoms and fertility concerns, as these factors persistently influence body image throughout the post-treatment phase, independent of time elapsed since diagnosis.

## CRediT authorship contribution statement

**Oshri Saar Sheffi:** Writing – review & editing, Writing – original draft, Investigation, Formal analysis, Data curation, Conceptualization. **Shani Paluch–Shimon:** Writing – review & editing, Supervision. **Gil Goldzwieg:** Writing – review & editing, Methodology, Formal analysis. **Shiran Klein Rotchild:** Investigation, Data curation, Conceptualization. **Michal Braun:** Writing – review & editing, Supervision, Formal analysis, Conceptualization.

## Ethical approval

This study received approval from the Ethics Committee of Tel Aviv Jaffa College (approval no. 2020050) and the Helsinki Committee of Shaarei Zedek Hospital (approval no. szmc-0302-20). Informed consent was obtained from each participant in the study.

## Data availability

The data that support the findings of this study are available from the corresponding author upon reasonable request.

## Funding sources

This research did not receive any specific grant from funding agencies in the public, commercial, or not-for-profit sectors.

## Declaration of competing interest

The authors declare no conflicts of interest.
